# Self-rated health status in relation to aircraft noise exposure, noise annoyance or noise sensitivity: the results of a cross-sectional study in France

**DOI:** 10.1186/s12889-020-10138-0

**Published:** 2021-01-10

**Authors:** Clémence BAUDIN, Marie LEFÈVRE, Patricia CHAMPELOVIER, Jacques LAMBERT, Bernard LAUMON, Anne-Sophie EVRARD

**Affiliations:** 1 Univ Lyon, Univ Gustave Eiffel, Ifsttar, Univ Lyon 1, Umrestte, UMR T_9405, Bron, France; 2 Institute for radiological protection and nuclear safety, Fontenay-aux-Roses, France; 3 Technical agency for information on hospital care, Lyon, France; 4 Univ Gustave Eiffel, Ifsttar, AME-DCM, Bron, France; 5 Currently retired, Villeurbanne, France; 6 Univ Gustave Eiffel, Ifsttar, TS2, Bron, France

**Keywords:** Epidemiology, Aircraft noise exposure, General health, Self-rated health status

## Abstract

**Background:**

Noise is a major public health issue because of its negative impacts on health, including annoyance, sleep disturbance, cardiovascular diseases and altered cognitive performance among children. Self-rated health status (SRHS) can be considered as a reliable indicator of quality of life, morbidity and mortality but few studies have considered SRHS in relation to aircraft noise exposure. The present study aims to investigate the association between this exposure and SRHS of people living near airports in France, and to consider the mediating or moderating role of aircraft noise annoyance and noise sensitivity in this association.

**Methods:**

This cross-sectional study included 1242 participants older than 18 and living near three major French airports. Information on their SRHS, aircraft noise annoyance, noise sensitivity and demographic, socioeconomic and lifestyle factors was collected during a face-to-face interview performed at home. Outdoor aircraft noise levels were estimated for each participant’s home address using noise maps. Logistic regressions with adjustment for potential confounders were used. The moderating and mediating effects of aircraft noise annoyance and noise sensitivity were investigated following Baron and Kenny’s recommendations.

**Results:**

A significant association was shown between aircraft noise levels and a fair/poor SRHS, only in men (OR=1.55, 95%CI 1.01–2.39, for a 10 dB(A)-increase in L_den_). This relationship was higher in men highly sensitive to noise (OR=3.26, 95%CI 1.19–8.88, for a 10 dB(A)-increase in L_den_). Noise sensitivity was associated with a fair/poor SRHS significantly in women (OR=1.74, 95%CI 1.12–2.68) and at the borderline of significance in men (OR=1.68, 95% CI 0.94–3.00), whereas aircraft noise annoyance was associated with a fair/poor SRHS only in men (OR=1.81, 95%CI 1.00–3.27).

**Conclusion:**

The present study confirms findings in the small number of available studies to date suggesting a positive association between aircraft noise levels and a fair/poor SRHS. These results also support the hypothesis that noise sensitivity would moderate this association. However, a mediating effect of annoyance cannot be excluded.

## Introduction

The understanding of the health effects of exposure to environmental noise has improved over the years. Noise is now considered as a major public health issue. Its negative impacts on health include annoyance [1], sleep disturbance [2, 3], cardiovascular diseases [4, 5] and altered cognitive performance among children [6]. According to the World Health Organization (WHO), more than a million healthy life years are lost every year due to environmental noise in Western Europe [7].

Self-rated health status (SRHS) has been shown to be a multidimensional indicator of general health that takes into account relevant information such as health and lifestyle practices, functional, coping and well-being dimensions [8, 9]. It may also reflect some aspects that are difficult to detect clinically, such as the early stage of a disease, disease severity, psychological and physiological resources, and social functioning [10]. There is much evidence that SRHS can be used as an indicator of quality of life, morbidity and mortality in the general population [11–17].

The latest WHO review on environmental noise in the European region, recently updated by Clark et al [18], highlights the fact that there is still scarce evidence of a relationship between aircraft noise and quality of life in European adult populations [6]. Although the relevance of SRHS has been shown, in particular to predict quality of life but not only, very few studies have investigated the effects of aircraft noise on this indicator for the population living near airports. Franssen et al used the single question: “How is your health in general?” [19]. They found that poor SRHS was associated with increased exposure to aircraft noise around Schiphol airport in Amsterdam. Two other studies used specific questionnaires to evaluate SRHS. In the Metropolitan Minnesota study, Meister et al found lower health scores on the Medical Outcome Study Short Form-36 Health Survey Scale (SF-36) [20] for the two neighborhoods that were exposed to higher levels of aircraft noise [21]. In the NORAH study, Schreckenberg et al showed significant negative correlation between mental and physical quality of life scores measured with a short version of SF-36, and aircraft noise levels around four airports in Germany [22].

According to the ISO’s definition, noise-induced annoyance corresponds to “one person’s individual adverse reaction to noise” where “the reaction may be referred to in various ways, including, for example, dissatisfaction, bother, annoyance and disturbance due to noise” [23]. According to Job in 1999, “noise sensitivity refers to the internal states (be they physiological, psychological [including attitudinal], or related to lifestyle or activities conducted) of any individual which increase their degree of reactivity to noise in general” [24]. Noise sensitivity is a personality trait, independent of noise levels, but has been shown to be a predictor of annoyance [25–27]. In contrast, noise annoyance has been found to be directly associated with noise levels [1, 28].

Both factors have been found to be directly associated to many health outcomes such as medication use, psychological distress, coronary heart disease and cardiovascular mortality [29–32]. However, very few studies have investigated the direct association between noise annoyance or noise sensitivity and SRHS. They have found that both road traffic noise annoyance and noise sensitivity were associated with SRHS. Nivison and Endresen found correlations between noise sensitivity (in women) or road traffic noise annoyance (in men) and health complaints [33]. Baliatsas et al showed that the higher the noise sensitivity, the worse the SRHS [34]. Ou et al and Riedel et al both found that road traffic noise annoyance was associated with lower SRHS [35, 36]. No studies have examined the association between aircraft noise annoyance and SRHS.

Previous studies suggest that both aircraft noise annoyance and noise sensitivity should be included in future analyses on the health effects of aircraft noise exposure. Tarnopolsky et al hypothesized that aircraft noise annoyance may be an intermediate step between aircraft noise exposure and self-reported symptoms [37]. Babisch underlined that one of the key issues for future research in this field is the role of the interaction between noise levels and annoyance in the relationship between noise levels and health outcomes [38]. Fyhri and Klæboe referred to the necessity of including noise sensitivity as a “crucial variable in research on noise–health relationships” [39]. Some studies have shown that these two factors may have a moderating or mediating role in the relationship between aircraft noise exposure and the use of medication, psychological distress, or hypertension [40–45]. However, no studies have examined the role of noise annoyance and noise sensitivity in the relationship between aircraft noise exposure and SRHS.

The present paper more specifically addresses the issue of SRHS in relation to aircraft noise exposure within the framework of the DEBATS (Discussion on the health effects of aircraft noise) research program, which is the first in France to investigate the effects of aircraft noise exposure on the health of populations living near airports. The question of whether increased levels of aircraft noise, noise annoyance, or noise sensitivity are associated with a weakening of SRHS is raised. Secondly, the potential moderating or mediating role of aircraft noise annoyance and noise sensitivity in the relationship between aircraft noise levels and SRHS was investigated. Finally, as women tend to judge their health more severely than men in France [46, 47], analyses were performed a priori for men and women separately.

## Methods

### Study population

The present cross-sectional analysis of the DEBATS study involved people who were over 18 years of age at the time of the interview, and living in the study area around the following three French international airports: Paris-Charles de Gaulle, Lyon Saint-Exupéry, and Toulouse-Blagnac [5]. The study area was defined based on existing noise contours near airports in terms of L_den_: < 50, 50–54, 55–59 and ≥60 dB(A). In the European Union (EU) directive 2002/49 relating to the assessment and management of environmental noise, L_den_ is defined as the weighted average of sound levels during the day (06:00 to 18:00), evening (18:00 to 22:00), and night (22:00 to 6:00), where the evening and night sound pressure levels have received a penalty of 5 dB(A) and 10 dB(A) respectively to reflect the additional sensitivity to noise during the evening and night. To ensure that a sufficient number of participants were exposed to aircraft noise levels above 60 dB(A) and below 50 dB(A), stratified random sampling was used to obtain contrasts in aircraft noise exposure based on these noise contour maps.

Participants were randomly selected from a phone directory, based on their address in the study area. Individuals were contacted by phone and included in the study when they agreed to participate. They signed and returned an informed consent by mail.

Data were collected on 1244 participants (549 men and 695 women) in 2013 [48]. All participants completed a questionnaire in a face-to-face interview at their place of residence. The questionnaire collected demographic and socioeconomic information, lifestyle factors including smoking, alcohol consumption, and physical activity, personal medical history in terms of sleep disturbance, cardiovascular disease, anxiety, depressive disorders, medication use, noise annoyance and noise sensitivity.

The analyses presented in this paper were performed on the 1242 participants (695 women and 547 men) who completed the information for all covariates included in the models.

### Exposure assessment

The “Integrated Noise Model” (INM) [49] is an internationally well-established computer model that evaluates the impact of aircraft noise near airports and produces noise contours for a given area. This model allows Paris Airports and the French Civil Aviation Authority to produce noise maps for the main French airports. These noise maps were used to estimate aircraft noise exposure at the participants’ place of residence, in front of the buildings. Outdoor aircraft noise exposure was assessed at intervals of 1 dBA for each participant by linking his/her home address to the noise contours using Geographical Information System (GIS) methods. Two noise indicators were used for the statistical analyses: L_den_ and L_Aeq,24h_. L_den_ was used to select participants. The L_Aeq,24h_ corresponds to average sound levels during the period of 24 h. Unlike L_den_, it is not weighted.

### Self-rated health status

Self-rated health status (SRHS) was measured with a single question in the face-to-face interview: “*In general, would you say that your health is excellent, good, fair, or poor?*”. In the statistical analyses, participants who reported a fair or poor SRHS were compared to those whose SRHS was good or excellent.

### Aircraft noise annoyance

Aircraft noise annoyance was assessed using the standard question recommended by the International commission on the biological effects of noise (Icben) using a five-point verbal response scale as follows [23]: “Thinking about the last 12 months when you are at home, how much does aircraft noise bother, disturb or annoy you? Extremely, very, moderately, slightly or not at all?”. Then, following the recommendations of Guski et al., the extremely or very annoyed participants were considered to be highly annoyed, and compared to the moderately, slightly and not at all annoyed participants, who were considered to be not highly annoyed [1].

### Noise sensitivity

Noise sensitivity was assessed by means of a five-point question in which participants were asked to estimate their own sensitivity: “Regarding noise in general, compared to people around you, do you think that you are: much less sensitive than, or less sensitive than, or as sensitive as, or more sensitive or much more sensitive than people around you?”. Participants who said they were much more or more sensitive than people around them were considered highly sensitive to noise. They were compared to participants who said they were much less, less or as sensitive as people around them who were considered not highly sensitive to noise.

### Confounding factors

The main potential confounding factors often mentioned in the literature about aircraft noise levels and health outcomes were obtained from the questionnaire and introduced into multivariate regression models: gender (dichotomous), age (six categories: 18–34; 35–44; 45–54; 55–64; 65–75; > 75 years old), country of birth (two categories: French-born/foreign-born), smoking habits (three categories: non/ex/occasional or daily smoker), number of people in the dwelling (four categories: 1; 2; 3; 4 and more), and household monthly income (three categories: < 2300 euros (2600 US$); 2300–4000 euros (2600–4500 US$); >=4000 euros (4500 US$)).

Alcohol consumption (four categories: no/light/moderate/heavy drinker) was initially included in the regression models. However, as it did not modify the odds-ratios (ORs) between aircraft noise levels and SRHS by more than 10%, it was not included in the final models.

Personal medical history has been shown to be a reliable measure of health outcomes and may also be considered as an indicator of SRHS [50, 51]. As it was highly correlated with SRHS, it is very likely that its inclusion in the models as a confounding factor would have resulted in over-adjustment.

### Statistical analysis

Logistic regression models were used to investigate the association between aircraft noise exposure/aircraft noise annoyance/noise sensitivity and SRHS. The M0 model (crude model) included in turn aircraft noise levels, aircraft noise annoyance and noise sensitivity as the main factor of interest. The M1 model included aircraft noise levels as the main factor of interest and potential confounders as covariates. The M2 model further included aircraft noise annoyance, while the M3 model further included noise sensitivity. The M4 model included aircraft noise annoyance as the main factor of interest, as well as potential confounding factors (without aircraft noise levels). The M5 model included noise sensitivity as the main factor of interest, as well as potential confounding factors (without aircraft noise levels).

Baron and Kenny’s recommendations were used to investigate the mediating role of aircraft noise annoyance and noise sensitivity in the relationship between aircraft noise levels and SRHS [52]. The results of the M1, M2 and M3 models were compared to assess a possible mediating effect of aircraft noise annoyance or of noise sensitivity. The moderating role of aircraft noise annoyance or noise sensitivity was investigated by including an interaction term between aircraft noise levels and aircraft noise annoyance (M6 model), and between aircraft noise exposure and noise sensitivity (M7 model) in the M1 model.

Statistical analyses were stratified by gender and performed separately for the two noise indicators (L_den_ and L_Aeq,24h_).

The linearity of the relationship between aircraft noise exposure and SRHS was tested using generalised additive models, including a smooth cubic function with linear and quadratic terms for aircraft noise exposure [53]. As the quadratic term was not significant in these models, the association with the continuous exposure variable per 10 dB(A) increase was finally estimated and presented in the present paper.

All statistical analyses were performed using SAS 9.3 (SAS Software [program] 9.3 version. USA: Cary North Carolina, USA 2011).

## Results

Table 1 presents the characteristics of the 1242 participants, stratified by categories of aircraft noise exposure (L_den_). The prevalence of a fair/poor SRHS in the DEBATS study population was 15% for men and 16% for women. It was similar among all aircraft noise categories for women, but varied from 9% in the < 50 dB(A) category to 20% in the 55–59 dB(A) category for men.

**Table 1:** Characteristics of the 1242 participants in the DEBATS study, stratified on aircraft noise exposure levels (L_den_ in dB(A))

	WOMEN (***N***=695)						MEN (***N***=547)					
	Aircraft noise levels (dB(A))				***Total*** ***N (%)***	***p (χ***^***2***^***)***	Aircraft noise levels (dB(A))				***Total*** ***N (%)***	***p (χ***^***2***^***)***
	< 50 *N*=164 (%)	50–54 *N*=166 (%)	55–59 *N*=190 (%)	≥ 60 *N*=175 (%)			< 50 *N*=152 (%)	50–54 *N*=141 (%)	55–59 *N*=123 (%)	≥ 60 *N*=131 (%)		
**Self-rated health status (SRHS)**						*0.93*						*0.08*
Excellent or good	139 (84.8)	140 (84.3)	162 (85.3)	145 (82.9)	586 (84.3)		138 (90.8)	120 (85.1)	98 (79.7)	110 (84.0)	466 (85.2)	
Fair/poor	25 (15.2)	26 (15.7)	28 (14.7)	30 (17.1)	109 (15.7)		14 (9.2)	21 (14.9)	25 (20.3)	21 (16.0)	81 (14.8)	
**Age**						***0.05***						*0.63*
18–34	48 (29.3)	33 (19.9)	41 (21.6)	30 (17.1)	152 (21.9)		20 (13.2)	21 (14.9)	18 (14.6)	15 (11.5)	74 (13.5)	
35–44	36 (22.0)	30 (18.1)	33 (17.4)	31 (17.7)	130 (18.7)		25 (16.4)	32 (22.7)	29 (23.6)	20 (15.3)	106 (19.4)	
45–54	26 (15.9)	36 (21.7)	42 (22.1)	29 (16.6)	133 (19.1)		40 (26.3)	30 (21.3)	32 (26.0)	30 (22.9)	132 (24.1)	
55–64	35 (21.3)	35 (21.1)	35 (18.4)	40 (22.9)	145 (20.9)		37 (24.3)	26 (18.4)	19 (15.4)	33 (25.2)	115 (21.0)	
65–74	15 (9.1)	21 (12.7)	21 (11.1)	33 (18.9)	90 (12.9)		26 (17.1)	24 (17.0)	18 (14.6)	26 (19.8)	94 (17.2)	
≥75	4 (2.4)	11 (6.6)	18 (9.5)	12 (6.9)	45 (6.5)		4 (2.6)	8 (5.7)	7 (5.7)	7 (5.3)	26 (4.8)	
**Country of birth**						***< 0.01***						*0.70*
France-born	150 (91.5)	146 (88)	150 (78.9)	149 (85.1)	595 (85.6)		128 (84.2)	121 (85.8)	99 (80.5)	110 (84.0)	458 (83.7)	
Foreign-born	14 (8.5)	20 (12.0)	40 (21.1)	26 (14.9)	100 (14.4)		24 (15.8)	20 (14.2)	24 (19.5)	21 (16.0)	89 (16.3)	
**Smoking**						*0.14*						*0.79*
Non smoker	76 (46.3)	91 (54.8)	111 (58.4)	101 (57.7)	379 (54.5)		64 (42.1)	67 (47.5)	59 (48.0)	56 (42.7)	246 (45.0)	
Ex-smoker	47 (28.7)	30 (18.1)	36 (18.9)	36 (20.6)	149 (21.4)		57 (37.5)	43 (30.5)	39 (31.7)	42 (32.1)	181 (33.1)	
Occasional or daily smoker	41 (25.0)	45 (27.1)	43 (22.6)	38 (21.7)	167 (24.0)		31 (20.4)	31 (22.0)	25 (20.3)	33 (25.2)	120 (21.9)	
**Number of people in the dwelling**						*0.39*						*0.32*
1	31 (18.9)	35 (21.1)	43 (22.6)	49 (28.0)	158 (22.7)		31 (20.4)	28 (19.9)	24 (19.5)	19 (14.5)	102 (18.6)	
2	60 (36.6)	53 (31.9)	60 (31.6)	66 (37.7)	239 (34.4)		57 (37.5)	46 (32.6)	38 (30.9)	56 (42.7)	197 (36.0)	
3	25 (15.2)	28 (16.9)	34 (17.9)	22 (12.6)	109 (15.7)		20 (13.2)	24 (17.0)	29 (23.6)	25 (19.1)	98 (17.9)	
≥ 4	48 (29.3)	50 (30.1)	53 (27.9)	38 (21.7)	189 (27.2)		44 (28.9)	43 (30.5)	32 (26.0)	31 (23.7)	150 (27.4)	
**Monthly household income**						*0.45*						*0.63*
≥ 4000 euros (4500 US$)	35 (21.3)	42 (25.3)	43 (22.6)	31 (17.7)	151 (21.7)		44 (28.9)	45 (31.9)	41 (33.3)	38 (29.0)	168 (30.7)	
2300–4000 euros (2600–4500 US$)	67 (40.9)	57 (34.3)	74 (38.9)	62 (35.4)	260 (37.4)		63 (41.4)	52 (36.9)	40 (32.5)	59 (45.0)	214 (39.1)	
< 2300 euros (2600 US$)	62 (37.8)	67 (40.4)	73 (38.4)	82 (46.9)	284 (40.9)		45 (29.6)	44 (31.2)	42 (34.1)	34 (26.0)	165 (30.2)	
**Noise sensitivity**						*0.52*						*0.68*
Not highly sensitive	110 (67.1)	105 (63.3)	133 (70.0)	111 (64.2)	459 (66.2)		112 (75.7)	100 (71.4)	92 (75.4)	101 (77.7)	405 (75.0)	
Highly sensitive	54 (32.9)	61 (36.7)	57 (30.0)	62 (35.8)	234 (33.8)		36 (24.3)	40 (28.6)	30 (24.6)	29 (22.3)	135 (25.0)	
**Aircraft noise annoyance**						***< 0.01***						***< 0.01***
Not highly annoyed	157 (95.7)	143 (86.1)	148 (77.9)	121 (69.1)	569 (81.9)		135 (88.8)	124 (87.9)	99 (80.5)	90 (68.7)	448 (81.9)	
Highly annoyed	7 (4.3)	23 (13.9)	42 (22.1)	54 (30.9)	126 (18.1)		17 (11.2)	17 (12.1)	24 (19.5)	41 (31.3)	99 (18.1)	

For women, some differences appeared between noise exposure categories for age and country of birth: women tended to be older and were more likely to have been born in a foreign country in the highest noise category (*p*< 0.05). For men, there was no difference between noise categories for the confounding factors. No difference in noise sensitivity was found between the noise categories for women and men (*p*=0.52 and *p*=0.68 respectively). For both genders, there were differences between the noise categories for annoyance due to aircraft noise: the more noise people were exposed to, the more annoyed they were (*p*< 0.0001). Severe noise annoyance was associated with increased aircraft noise levels in both men and women, with an OR=2.43, 95% confidence intervals (CI) 1.67–3.55 in men and OR=3.44, 95% CI 2.36–5.02 in women, for a 10 dB(A)-increase in L_den_.

Table 2 shows the ORs and their 95% CIs for a fair/poor SRHS in relation to aircraft noise exposure, aircraft noise annoyance and noise sensitivity. No relationship was found between aircraft noise exposure and a fair/poor SRHS for women, regardless of the noise indicator, and the inclusion of confounding factors in the model (M0 and M1 models). In contrast, the association between aircraft noise exposure and a fair/poor SRHS was statistically significant in men, regardless of the noise indicator, and the inclusion of confounding factors in the model (OR=1.52, 95% CI 1.01–2.29 in M0 model, and OR=1.55, 95% CI 1.01–2.39 in M1 model, for a 10 dB(A)-increase in L_den_).

**Table 2:** Odds-ratios for a fair/poor self-rated health status (SRHS) in relation to aircraft noise exposure, aircraft noise annoyance and noise sensitivity

		WOMEN		MEN	
		OR	95% CI	OR	95% CI
M0 model	LA_eq. 24h_^a^	1.02	(0.69–1.50)	**1.67**	**(1.06–2.62)**
	L_den_^a^	1.06	(0.74–1.51)	**1.52**	**(1.01–2.29)**
	Aircraft noise annoyance^b^	1.25	(0.76–2.08)	**1.88**	**(1.09–3.26)**
	Noise sensitivity^c^	**1.61**	**(1.06–2.44)**	1.35	(0.80–2.27)
M1 model	LA_eq. 24h_^a^	0.97	(0.64–1.45)	**1.69**	**(1.05–2.74)**
	L_den_^a^	1.01	(0.69–1.45)	**1.55**	**(1.01–2.39)**
M2 model	LA_eq. 24h_^a^	0.95	(0.62–1.44)	1.56	(0.95–2.56)
	Aircraft noise annoyance^b^	1.11	(0.64–1.91)	1.60	(0.87–2.94)
	L_den_^a^	0.99	(0.67–1.45)	1.44	(0.92–2.24)
	Aircraft noise annoyance^b^	1.09	(0.63–1.88)	1.61	(0.87–2.97)
M3 model	LA_eq. 24h_^a^	0.97	(0.65–1.46)	**1.75**	**(1.08–2.85)**
	Noise sensitivity^c^	**1.74**	**(1.12–2.68)**	1.73	(0.97–3.11)
	L_den_^a^	1.01	(0.69–1.46)	**1.62**	**(1.04–2.52)**
	Noise sensitivity^c^	**1.73**	**(1.12–2.68)**	1.73	(0.97–3.11)
M4 model	Aircraft noise annoyance^b^	1.08	(0.64–1.83)	**1.81**	**(1.00–3.27)**
M5 model	Noise sensitivity^c^	**1.74**	**(1.12–2.68)**	1.68	(0.94–3.00)

^*a*^ *Per 10 dB(A) increase*

^*b*^ *Odds ratio for highly annoyed people compared to those who were not highly annoyed*

^*c*^ *Odds ratio for people highly sensitive to noise compared to those who were not highly sensitive to noise*

*M0 model included in turn noise levels, aircraft noise annoyance and noise sensitivity*

*M1 model included noise levels, age, country of birth, smoking, number of people in the dwelling, monthly household income*

*M2 model included aircraft noise annoyance, noise levels, age, country of birth, smoking, number of people in the dwelling, monthly household income*

*M3 model included noise sensitivity, noise levels, age, country of birth, smoking, number of people in the dwelling, monthly household income*

*M4 model included aircraft noise annoyance, age, country of birth, smoking, number of people in the dwelling, monthly household income*

*M5 model included noise sensitivity, age, country of birth, smoking, number of people in the dwelling, monthly household income*

*Bold values are statistically significant (p<0.05)*

When aircraft noise annoyance was included in the M1 model, the association between aircraft noise levels and a fair/poor SRHS in men became lower and not significant (OR=1.44, 95% CI 0.92–2.24, for a 10 dB(A)-increase in L_den_) (M2 model). When noise sensitivity was included in the M1 model, the association between aircraft noise levels and a fair/poor SRHS in men remained similar (M3 model).

A significant association was observed between aircraft noise annoyance and a fair/poor SRHS only in men (OR=1.81, 95% CI 1.00–3.27 for highly annoyed men vs. not highly annoyed men) (M4 model). A significant association was observed between noise sensitivity and a fair/poor SRHS in women (OR=1.74, 95% CI 1.12–2.68 for highly sensitive women vs. not highly sensitive women) (M5 model). This association was not significant for men, but at the borderline of significance and of the same order of magnitude as for women (OR=1.68, 95% CI 0.94–3.00 for highly sensitive men vs. not highly sensitive men) (M5 model).

Table 3 presents the ORs for the relationship between aircraft noise exposure and a fair/poor SRHS in highly annoyed vs not highly annoyed men and women, and in highly noise sensitive vs not highly noise sensitive men and women. This association was higher, but still not significant, in highly annoyed women compared to those not highly annoyed (M6 model). However, this difference was not statistically significant (*p*=0.31 for the L_den_). The association between aircraft noise exposure and a fair/poor SRHS was significantly higher in men who were highly sensitive to noise (OR=3.26, 95% CI 1.19–8.88, for a 10 dB(A)-increase in L_den_) than for those who were not (OR=1.19, 95% CI 0.71–2.00, for a 10 dB(A)-increase in L_den_) (*p*< 0.05) (M7 model).

**Table 3:** Odds-ratios for a fair/poor self-rated health status (SRHS) in relation to aircraft noise exposure in highly annoyed/sensitive to noise men and women compared to those who are not

		M6 model					M7 model				
		Not highly annoyed people		Highly annoyed people		p _(interaction)_^a^	People not highly sensitive to noise		People highly sensitive to noise		p_(interaction)_^b^
		OR	95% CI	OR	95% CI		OR	95% CI	OR	95% CI	
WOMEN	LA_eq. 24h_^a^	0.81	(0.51–1.29)	1.40	(0.38–5.24)	0.25	1.00	(0.58–1.71)	0.89	(0.46–1.70)	0.51
	L_den_^a^	0.88	(0.58–1.33)	1.36	(0.38–4.89)	0.31	1.03	(0.63–1.69)	0.96	(0.53–1.71)	0.53
MEN	LA_eq. 24h_^a^	1.53	(0.86–2.73)	1.54	(0.47–4.99)	0.74	1.24	(0.69–2.21)	**3.72**	**(1.28–10.8)**	**0.04**
	L_den_^a^	1.43	(0.85–2.39)	1.35	(0.45–4.03)	0.76	1.19	(0.71–2.00)	**3.26**	**(1.19–8.88)**	**0.05**

*M6 and M7 models included aircraft noise levels, age, country of birth, smoking, the number of people in the dwelling, and monthly household income*

^*a*^ *p-value for the interaction between aircraft noise levels and aircraft noise annoyance*

^*b*^ *p-value for the interaction between aircraft noise levels and noise sensitivity*

*Bold values are statistically significant (p< 0.05)*

## Discussion

The DEBATS study was the first in France and one of the few in Europe to investigate the relationship between aircraft noise exposure and SRHS of populations living near airports.

The results of the present study suggest an association between aircraft noise exposure and a fair/poor SRHS. It seems to confirm the findings of previous studies. The study conducted among residents of Amsterdam Schiphol airport was the most similar to the DEBATS study in terms of methodology [19]. It evaluated the impact of aircraft noise on SRHS using a single question “How is your health in general?” and a 13-item Dutch validated questionnaire (VOEG) consisting of a list of health complaints. The results of this study showed a significant association between aircraft noise exposure and SRHS as assessed by the single question (OR=1.23, 95% CI 1.04–1.46), or as assessed by health complaints using the VOEG questionnaire (OR=1.21, 95% CI 1.02–1.43), in both cases for a 10 dB(A)-increase in L_den_. Other studies examined the effects of aircraft noise exposure on SRHS using validated questionnaires. A neighborhood study in metropolitan Minnesota showed that general health scores on the MOS-36 Health Survey Scale were significantly lower in the two most exposed neighborhoods [21].

In the present study, the association between aircraft noise exposure and a fair/poor SRHS was positive and significant for men, but not for women. This gender difference may be due to some unmeasurable confounding factors that would be more prevalent in men than in women, e.g. coping or ability to accept pain. However, it is consistent with the results of Halonen et al in Finland who showed an association between exposure to road traffic noise and an increased risk of a poor SRHS in men (OR=1.58, 95% CI 1.14–2.21 for noise levels above 60 dB(A) compared to noise levels below 45 dB(A) in L_den_) [54]. It is also consistent with a previous result obtained in the DEBATS study, which suggests that aircraft noise exposure may increase the risk of hypertension in men, but not in women [48]. Babisch et al have proposed a mechanism by which noise exposure induces a disturbance or annoyance, which leads to a stress response, activating the endocrine system, and resulting in physiological health problems [38]. A complementary hypothesis that the activation of the endocrine system may differ according to gender is suggested [55, 56]. This would explain the gender-differences in the results of several studies on the effects of traffic noise exposure on cardiovascular disease [48, 57–59]. In addition, since some studies have shown that endocrine distress can lead to psychological symptoms, this would also explain the gender-difference observed in this study between aircraft noise exposure and SRHS.

When the analyses were limited to the 991 participants who had been living at their home address for at least five years, the ORs and *p*-values in men were higher for all noise indicators. The association remained non-significant for women. These results support the hypothesis that the accumulation of aircraft noise exposure over time leads to a higher risk of a fair/poor SRHS.

This study showed a significant association between aircraft noise annoyance and a fair/poor SRHS. It seems to confirm the findings of two previous studies [35, 36]. This association was positive and statistically significant in men but not in women (OR=1.81, 95% CI 1.00–3.27, and OR=1.08, 95% CI 0.64–1.83, respectively, for men or women who were highly annoyed vs. those who were not highly annoyed). This gender difference has never been considered in SRHS and might also be due to unmeasurable confounding factors or to gender-differences in the activation of the endocrine system, as mentioned earlier. When aircraft noise annoyance was included as a covariate in the M1 model (M2 model), the association between aircraft noise levels and a fair/poor SRHS became lower and not significant in men (from OR=1.55, 95% CI 1.01–2.39 in M1 to OR=1.44, 95% CI 0.92–2.24 in M2, with a 10 dB(A)-increase in L_den_). As well as aircraft noise levels were significantly associated with aircraft noise annoyance [28], and aircraft noise annoyance was significantly associated with SRHS, according to Baron and Kenny, aircraft noise annoyance would have a mediating role in the relationship between aircraft noise levels and SRHS [52]. Finally, the ORs for a fair/poor SRHS in relation to aircraft noise exposure were higher for highly annoyed women (OR=1.36, 95% CI 0.38–4.89 for a 10 dB(A)-increase in L_den_) than for not highly annoyed women (OR=0.88, 95% CI 0.58–1.33 for a 10 dB(A)-increase in L_den_), supporting the hypothesis of a non-significant moderating role of aircraft noise annoyance in this relationship. This difference was not significant, probably due to a lack of statistical power. Indeed, the detection of the moderating role of annoyance with the model including an interaction term would require approximately four times as many women as compared to the model without the interaction.

This study also found an association between noise sensitivity and a fair/poor SRHS. It seems to confirm the findings of a previous study [34]. This association was significant in women and at the borderline of significance in men (OR=1.74, 95% CI 1.12–2.68, and OR=1.68, 95% CI 0.94–3.00, in women and men respectively, for participants who were highly sensitive to noise vs. those who were not highly sensitive to noise). When noise sensitivity was included in the M1 model with aircraft noise levels (M3 model), the results remained very similar, suggesting that noise sensitivity cannot be considered as a mediator in the relationship between noise levels and SRHS. However, the association between aircraft noise exposure and a fair/poor SRHS observed only in men was significantly higher in men who considered themselves highly sensitive to noise (OR=3.26, 95% CI 1.19–8.88, for a 10 dB(A)-increase in L_den_) than in men not highly sensitive to noise (OR=1.19, 95% CI 0.71–2.00, for a 10 dB(A)-increase in L_den_). This result suggests that noise sensitivity would have a moderating role in this association.

Nevertheless, selection bias, uncontrolled or residual confounding, recall bias, and exposure misclassification should be considered.

Indeed, selection bias could occur regarding the characteristics of participants compared to people who refused to participate. Forty percent of them agreed to respond to a short questionnaire in order to establish a brief demographic and socioeconomic profile of non-participants. Participants were slightly older and were more likely to be in managerial or higher-level professional occupations than non-participants [32]. Nevertheless, the representativeness of these non-participants compared to all people who refused to participate, and the representativeness of the study population compared to all people living near an airport in France can be noted. However, due to a lack of information, it was not possible to characterize these populations in the present study.

Moreover, the possible adverse health effects of aircraft noise could have led to a lower proportion of sensitive people among those living near airports, especially in the noisiest areas. People who considered themselves particularly vulnerable to noise may be reluctant to live in noisy conditions. There is little information available to judge whether this has happened. However, if it did occur, it would have led to an underestimation of the association between aircraft noise exposure and SRHS in this study.

The assessment of a very large number of covariates in the questionnaire made it possible to evaluate a large number of possible confounding factors and to ensure the stability of the results. However, uncontrolled or residual confounding cannot be excluded. In the vicinity of airports, residential property values are reduced, in particular due to aircraft noise [60–62]. This might lead to an over-representation of local residents with low socio-economic status and subsequently to poorer SRHS [63]. However, this study collected information on the socio-economic status of participants and the results presented here were controlled for the effect of monthly household income (used as a proxy of the socioeconomic status) on SRHS.

Aircraft noise exposure was estimated at the place of residence of the participants. As a result, misclassification of aircraft noise exposure could also occur, particularly regarding daytime exposure, as participants were more likely to be outside their homes during the day than at night. Noise in the workplace in particular could be a factor of a fair/poor SRHS or could interact with residential noise. Unfortunately, no information was available on participants’ exposure to daytime noise outside their home, particularly in the workplace. However, classification of exposure would probably not depend on SRHS. Therefore, such a non-differential misclassification would have induced an appreciable downward bias, if there is a true association between aircraft noise exposure and SRHS.

The present study had a specific strength in the assessment of noise exposure. Indeed aircraft noise levels were estimated for each participant by means of modelled noise calculations produced by the French Civil Aviation Authority using INM software [49]. These modelled noise levels were validated by comparison with measurements made by permanent stations or during specific campaigns around airports. The differences were mainly between 0.5 and 1.5 dB (A) in terms of L_den_.

While the WHO Environmental Noise Guidelines for the European Region referred to the L_den_ as the exposure metrics of choice when considering health outcomes [64], L_Aeq,24h_ was also used in the present analyses. Indeed, the penalties included in the L_den_ take into account the level of annoyance due to aircraft noise in the evening and at night. As one of the main objectives of this study was to investigate the role of aircraft noise annoyance in the relationship between aircraft noise levels and SRHS, the use of the L_den_ would have biased the results downwards. This is actually not the case since the results obtained in this study were the same whether L_den_ or L_Aeq,24h_ was used.

In addition, the assessment of SRHS by a single question would have the same (or even greater) reliability as specific questions on functional capacity, number of chronic conditions, or psychological well-being [65]. In the study by Lundberg et al, good reliability in terms of weighted kappa values of SRHS was shown on a test-retest basis among the different age and gender subgroups studied. The SRHS is also a valid and effective measure of general health status: the concurrent validity of SRHS for physical and mental health has recently been demonstrated for both genders in 19 European countries [66]. Although many studies have demonstrated the validity, reliability and benefit of using a single question to assess SRHS, very few studies have measured SRHS with a single question in the literature [19, 54] while others have used questionnaires or symptoms reports. Moreover, there is still no standard wording for this question, with differences in wording and scoring of the question hampering comparisons between studies.

SRHS is widely used to measure the general health status of populations. However, it is not clear how the health assessment process can vary according to several demographic and socioeconomic characteristics, including gender, age, ethnicity, and education. The types of health factors that participants consider and how they take these health factors into account when assessing their health are not well-defined [67].

Aircraft noise annoyance and noise sensitivity in relation to SRHS, or their moderating or mediating role in the relationship between aircraft noise levels and a fair/poor SRHS have not been often studied in the literature. This study has been successful in providing more information on these relationships, and has suggested a significant role for noise annoyance and noise sensitivity, as other studies have done for other outcomes, recommending that these factors be considered in studies about health effects of noise. Further research is therefore needed to deepen our understanding of this process and the causal pathway between noise exposure and a fair/poor SRHS and its subsequent physiological health effects.

## Conclusion

The DEBATS study was the first in France and one of the few in Europe to investigate the relationship between aircraft noise exposure and self-rated health status of populations living near airports. After adjusting for potential confounding factors, the results suggest that the more men were exposed to aircraft noise, the more likely they were to report a fair/poor SRHS. Furthermore, the results support the hypothesis that noise sensitivity would have a moderating role in this association, which would not be the case for noise annoyance. However, the mediating effect of annoyance cannot be excluded. Nevertheless, further studies are needed to confirm this conclusion.

## Data Availability

The data that support the findings of this study were collected and used after the approval of two national authorities in France, the French Advisory Committee for Data Processing in Health Research and the French National Commission for Data Protection and the Liberties, and so are not publicly available. Data are however available from the authors upon reasonable request and with permission of the French National Commission for Data Protection and the Liberties.

## Abbreviations

95% CI: 95% confidence interval
DEBATS: Discussion on the health effects of aircraft noise
EU: European Union
GIS: Geographical Information System
ICBEN: International Commission on the Biological Effects of Noise
INM: Integrated Noise Model
ISO: International Organization for Standardization
OR: Odds-ratio
SRHS: Self-rated health status
VOEG: A13-item Dutch validated questionnaire
WHO: World Health Organization
